# Simple Audit of Trainer/Trainee Consultation Profiles as a Method of Assessing Whether Non-Structured Surgeries Give Adequate Training

**Published:** 1988-08

**Authors:** F. D. Skerrett, Michael Black, Regan Wooding, Ciarnan Kelly

**Affiliations:** The Health Centre Eastcliffe Road, Par. Trainer Cornwall VTS; The Surgery, St. Mawes, Cornwall. Trainee 1983; 40 Nelson Street, Norwich. Trainee 1985; 4 Fuschia Park, Renmore, Galway. Trainee 1986


### Simple Audit of Trainee/Trainer Consultation Profiles as a Method of Assessing Whether Non Structured Surgeries Give Adequate Training

**F. D. Skerrett**
The Health Centre Eastcliffe Road, Par. Trainer Cornwall VTS.
**Michael Black**
The Surgery, St. Mawes, Cornwall. Trainee 1983
**Regan Wooding**
40 Nelson Street, Norwich. Trainee 1985
**Ciarnan Kelly**
4 Fuschia Park, Renmore, Galway. Trainee 1986.

During the year 1983/84 M. B. was attached to our training practice at Fowey. As one of his projects he analysed the consultations he held during his trainee year. He devised his own classification of diseases and the total number of cases seen in the year was as follows:

**Table 1**

| | |
|---|---|
| Respiratory | 610 |
| Ortho/Trauma | 306 |
| Neur/Sens. | 273 |
| C.V.S. | 265 |
| Gastro. Int. | 192 |
| Psychiatric | 191 |
| Prevent/Soc | 158 |
| Non specific | 132 |
| Genito/Urin. | 117 |
| Infection | 056 |
| Endocrine | 055 |
| Neoplasia | 025 |
| Total | 2380 |

This suggested that MB had seen a wide spectrum of general practice during his year as a trainee. FDS was asked to take part in the audit for one week when the cases seen were also divided into acute and chronic conditions. During this week FDS saw 88 patients, 42 with acute conditions and 46 cases with chronic illness whereas MB saw 67 patients (38 acute and 29 chronic). The analysis of the cases are shown in Table 2.

This audit of trainer and trainee consultations over 1 week suggested that MB was experiencing a similar spread of cases as his trainer. This implied that without monitoring or channelling of cases a trainee would appear to be exposed to and gain adequate experience of "normal" general practice. The length of the audit with the small numbers involved was felt to be inadequate to prove that our method of non selection or channelling of cases gave adequate work experience. It was decided to repeat the trainer-trainee audit over a year and to use this audit as a method of introducing the microprocessor as a practice tool and by using the machine give the trainee experience in storing and manipulating data.

Correspondence to F. D. Skerrett, The Health The Health Centre, Par. Cornwall

### METHODS

The cases seen by the trainee and the trainer were classified into disease classes and were recorded for the same week at intervals over the trainee year 1984/85(RW). In all seven weeks were audited during this period. The audit was expanded in 1985/86 so that a week's analysis took place for each month of the year and at CK's request an age of patient consulting was added. The audit was based on the old RCGP codes which were used in Update to analyse practice consultation with the addition of a special category for consultations whose content was deemed to be of a purely social nature. A list of these categories is shown below in Table 3. Although these are gross in comparison with the latest RCGP classification of diseases they were thought to be adequate for our purposes. The decision to conduct the audit over the year was made to ensure that seasonal variations were assessed to further validate our procedure. A simple spread sheet was used to store data (Mini Office 11).

### RESULTS

The results are shown below in tabular form. The figures have been expresed as percentages of the total number of patients seen. The age audit is shown in Table 4 and shows the expected result that the trainee was seeing rather more of the younger population although there was a marked difference between the early months and the later months when the figures for October are compared with the total seen over the years by FDS.

**Table 2**

| | *CVS* | *Orth* | Psych | *G.I.T.* | *G.U.S.* | *Derm* | *Neuro* | *Endo* | *Infec* | *Resp* |
|---|---|---|---|---|---|---|---|---|---|---|
| MB | 7 | 7 | 3 | 4 | 5 | 5 | 9 | 3 | 3 | 19 |
| FDS | 14 | 11 | 10 | 9 | 9 | 8 | 8 | 6 | 6 | 10 |

**Table 3**

| *Diagnosis* | *RCGP NO* | 1985 *R.W.* | *F.D.S.* | 1986 *C.K.* | *F.D.S.* |
|---|---|---|---|---|---|
| Communicable Dis. | 1–43 | 3.8% | 0.27 | 1.25 | 0.16 |
| Neoplasms | 50–82 | 0.7 | 1.2 | 0.23 | 2.03 |
| Allergy/Endocrine | 85–105 | 3.1 | 2.35 | 2.94 | 3.4 |
| Blood | 110–122 | 1.0 | 1.1 | 0.1 | 0.70 |
| Personal/mental | 124–151 | 6 | 3.8 | 2.0 | 5.6 |
| C.N.S. | 155–207 | 3 | 2.8 | 4.63 | 2.9 |
| Circulatory | 209–307 | 6.9 | 20.5 | 10.97 | 20.4 |
| Respiratory | 240–272 | 24.1 | 18.7 | 30.4 | 17.76 |
| Digestion | 274–308 | 6.1 | 4.3 | 7 | 5.86 |
| Genito-urinary | 310–344 | 8.8 | 8.3 | 6.56 | 7.20 |
| Pregnancy | 345–366 | 4.6 | 10.4 | 0.8 | 7.3 |
| Skin & Connective | 368–399 | 12.8 | 7.2 | 13.57 | 7.3 |
| Bones & Joints | 404–427 | 9.7 | 9.4 | 10.7 | 12.6 |
| Congenital Malform | 429–439 | 0 | 0 | 0 | 0 |
| Disease/Infancy | 440–451 | 0.90 | 0.4 | 0.9 | 0.5 |
| Symptoms | 454–464 | 3.3 | 3.8 | 2.0 | 2.4 |
| Trauma | 467–464 | 1.3 | 1.66 | 0.9 | 0.4 |
| Prophylaxis | 500–586 | 1.2 | 0.55 | 2.7 | 0.81 |
| Social | | 2.3 | 3.0 | 2.15 | 2.8 |
| Total | | 462 | 722<br>1184 | 884 | 1227<br>2111 |

**Table 4**

| | *CK TOTAL* % | *FDS TOTAL* % | *CK OCT* % | *FDS OCT* % |
|---|---|---|---|---|
| 0–4y 11mths | 12.3 | 4.8 | 4.6 | 2.0 |
| 5y–9y 11mths | 10.0 | 2.7 | 6.25 | 1.0 |
| 10y–14y 11mths | 5.7 | 2.7 | 6.25 | 3.0 |
| 15y–19y 11mths | 8.0 | 4.3 | 7.8 | 0.0 |
| 20y–24y 11mths | 4.7 | 5.0 | 4.6 | 2.0 |
| 25y–29y 11mths | 6.9 | 5.8 | 0.0 | 9.0 |
| 30y–34y 11mths | 7.25 | 5.3 | 14.0 | 3.0 |
| 35y–39y 11mths | 7.5 | 0.5 | 9.3 | 5.0 |
| 40y-44y 11mths | 4.3 | 4.6 | 6.25 | 7.0 |
| 45y–49y 11mths | 5.7 | 4.8 | 9.3 | 2.0 |
| 50y–54y 11mths | 6.5 | 6.2 | 9.3 | 8.0 |
| 55y–59y 11mths | 3.6 | 11.0 | 9.3 | 9.0 |
| 60y–64y 11mths | 6.3 | 9.6 | 1.5 | 7.0 |
| 65y–69y 11mths | 3.5 | 7.6 | 2.4 | 8.0 |
| 70y–74y 11mths | 3.0 | 10.3 | 2.4 | 19.0 |
| 75y–79y 11mths | 2.2 | 6.9 | 4.6 | 8.0 |
| 80y–84y 11mths | 1.8 | 2.8 | 1.5 | 1.6 |
| 85y–98y 11mths | 0.4 | 0.3 | 1.5 | 0.0 |
| 90y plus | 0.14 | 0.3 | 0.0 | 1.0 |
| Total | 716 | 1011 | 64 | 100 |

### DISCUSSION

This audit of consultations undertaken by trainee and trainer has provided us with much valuable data. From the figures it is interesting to note that the trainer appeared to see a similar percentage of cases from year to year. Taken overall the trainees see a similar pattern and possibly an adequate spread of cases over the year for us to feel that the non-selection of surgery cases enable the trainee to obtain a fair insight into practice consultation habits. Comparison with the paper published in the RCGP Journal (1) by Flemming showed a very similar pattern of cases seen but did not set out with the object of comparison between trainer and trainee. Comparison with the National Morbidity Survey and the General Household Survey did however show some variation particularly for those groups which increase disproportionately with age (e.g. CVS., respiratory system and arthritis.) This was to be expected in Cornwall with its greater than expected elderly population structure. This audit has implications for practice logistics because if our system gave a good exposure and fair training then there would be saving in receptionist and doctor time in arranging the surgeries. It also gave practice information as to the areas in which the training experience was not adequate. Antenatal and pregnancy problems accounted for 4.5% for RW and 0.8% for CK as compared to the trainer who saw 10.4% and 7.3% respectively during the respective years. This justified a change in the trainees timetable to give a better opportunity for experience in this field. The age audit confirmed what we had expected but it is interesting to see the figures of the year for CK compared to the figures for his last month when he appeared to have changed the age pattern to resemble more that seen by FDS. Any

*continued on p53*

*continued from p39*

### Simple Audit of Trainer/Trainee Consultations concern about lack of experience in the problems of the elderly we felt could be adjusted by home visit selection and by encouragement in a limited and self selected "own list".

Two major opportunities were not taken by this audit. The first was not to extend the work of the original audit by MB to look at the numbers of the acute as opposed to the chronic cases seen by the trainee. Secondly, a comparison of the sex ratio of cases seen by male as opposed to female trainees and the trainer and this may have made an interesting paper. (Fortunately this has become of more academic interest with the advent of a lady partner). Whether the use of the microprocessor for storing and manipulation for this audit will have lasting educational effect on the trainees will be reviewed in the future. MB introduced the practice to the computer and we hope that the extended audit will have its intended effect but FDS has some concern over this aspect of anticipated change in attitude!

### REFERENCES

1. FLEMMING, D. M. RCGP Journal. Vol **36**, No. 286. p.212
2. 1981/2 National Morbidity Survey Table 8.8 p.28

